# Supplementary material for: Female Psammomys obesus Are Protected from Circadian Disruption-Induced Glucose Intolerance, Cardiac Fibrosis and Adipocyte Dysfunction
Source: Int J Mol Sci. 2024 Jul 1;25(13):7265. doi: 10.3390/ijms25137265 (PMC11242371; doi:10.3390/ijms25137265)
Supplement: Supplementary file 1 [file ijms-25-07265-s001.zip › ijms-3063784-supplementary.pdf]

Supplemental Figure S1

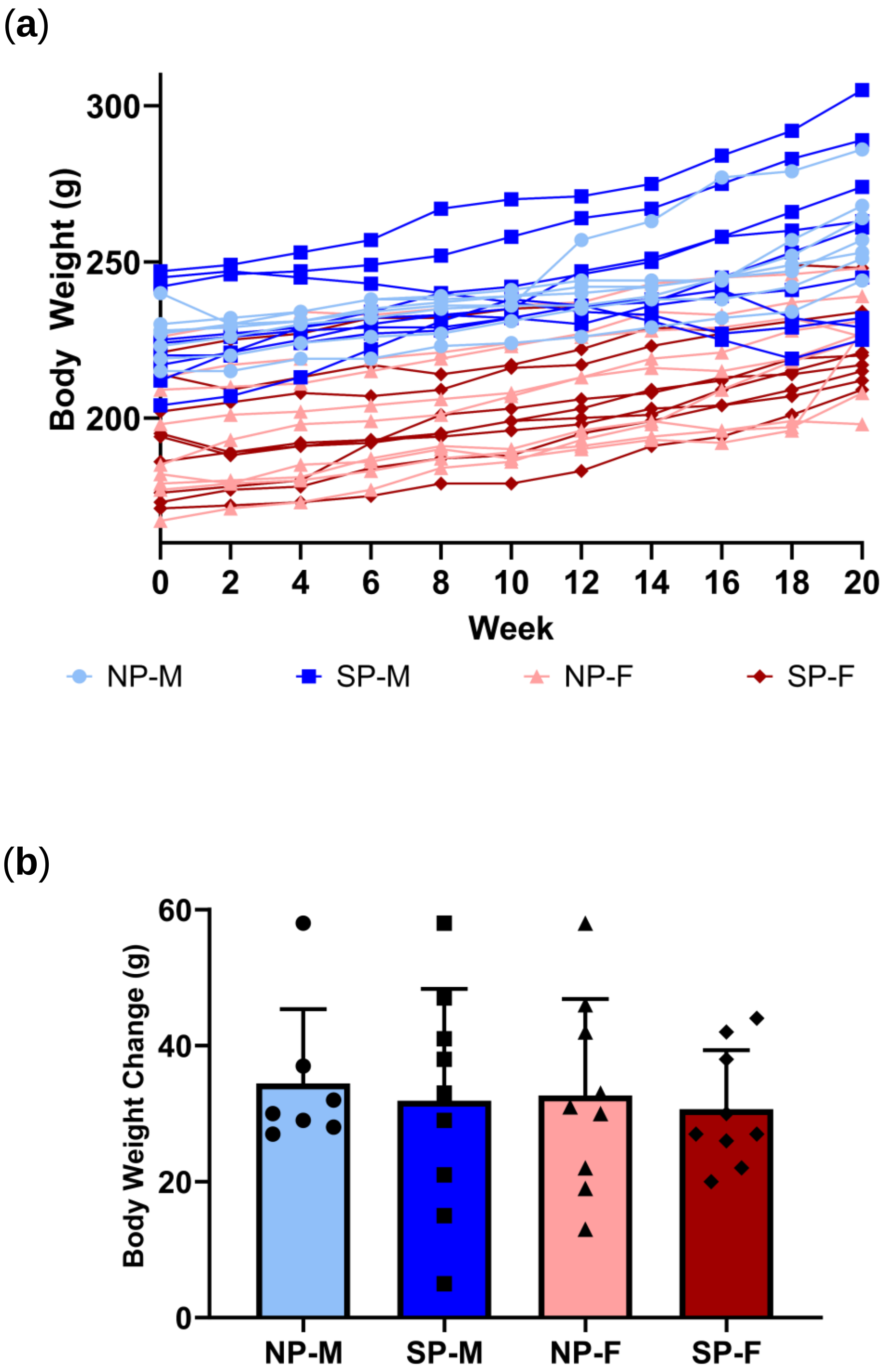

Supplemental Figure S1: Timeline of body weights. Male and female *P. obesus* were exposed to neutral (12 hr light:12 hr dark) or short (SP, 5 hr light:19 hr dark) photoperiods and a high energy diet for 20 weeks. (**a**) Body weights were measured for the duration of the study. (**b**) Body weight change. NP-M: neutral photoperiod males, SP-M: short photoperiod males, NP-F: neutral photoperiod females, SP-F: short photoperiod females.

Supplemental Figure S2

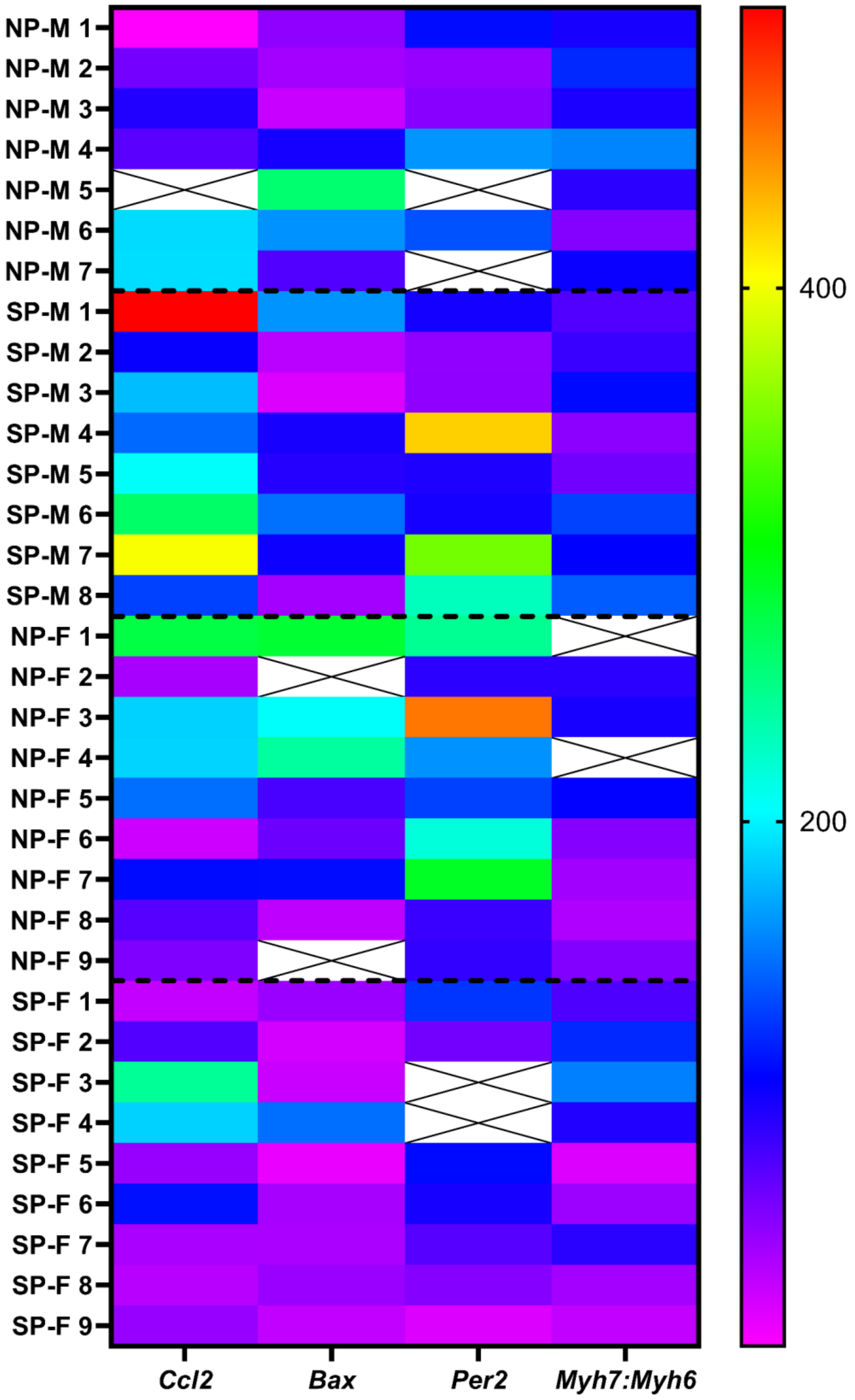

Supplemental Figure S2: Differential cardiac gene changes with short photoperiod exposure in males and females. Male and female *P. obesus* were exposed to neutral (12 hr light:12 hr dark) or short (SP, 5 hr light:19 hr dark) photoperiods and a high energy diet for 20 weeks. Expression heatmap showing differential cardiac expression of *Ccl2*, *Bax*, *Per2* and *Myh7:Myh6* mRNA. NP-M: neutral photoperiod males, SP-M: short photoperiod males, NP-F: neutral photoperiod females, SP-F: short photoperiod females.

Supplemental Figure S3

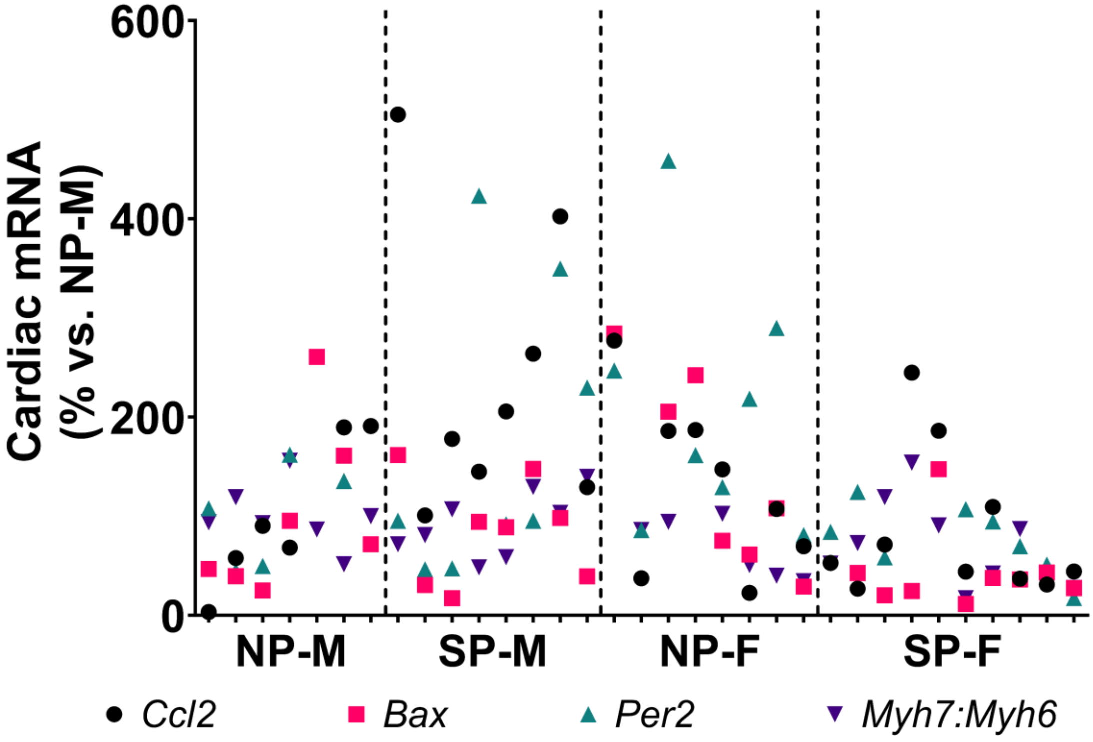

Supplemental Figure S3: Differential cardiac gene changes with short photoperiod exposure in males and females. Male and female *P. obesus* were exposed to neutral (12 hr light:12 hr dark) or short (SP, 5 hr light:19 hr dark) photoperiods and a high energy diet for 20 weeks. XY plot showing differential cardiac expression of *Ccl2*, *Bax*, *Per2* and *Myh7:Myh6* mRNA. NP-M: neutral photoperiod males, SP-M: short photoperiod males, NP-F: neutral photoperiod females, SP-F: short photoperiod females.

Supplemental Figure S4

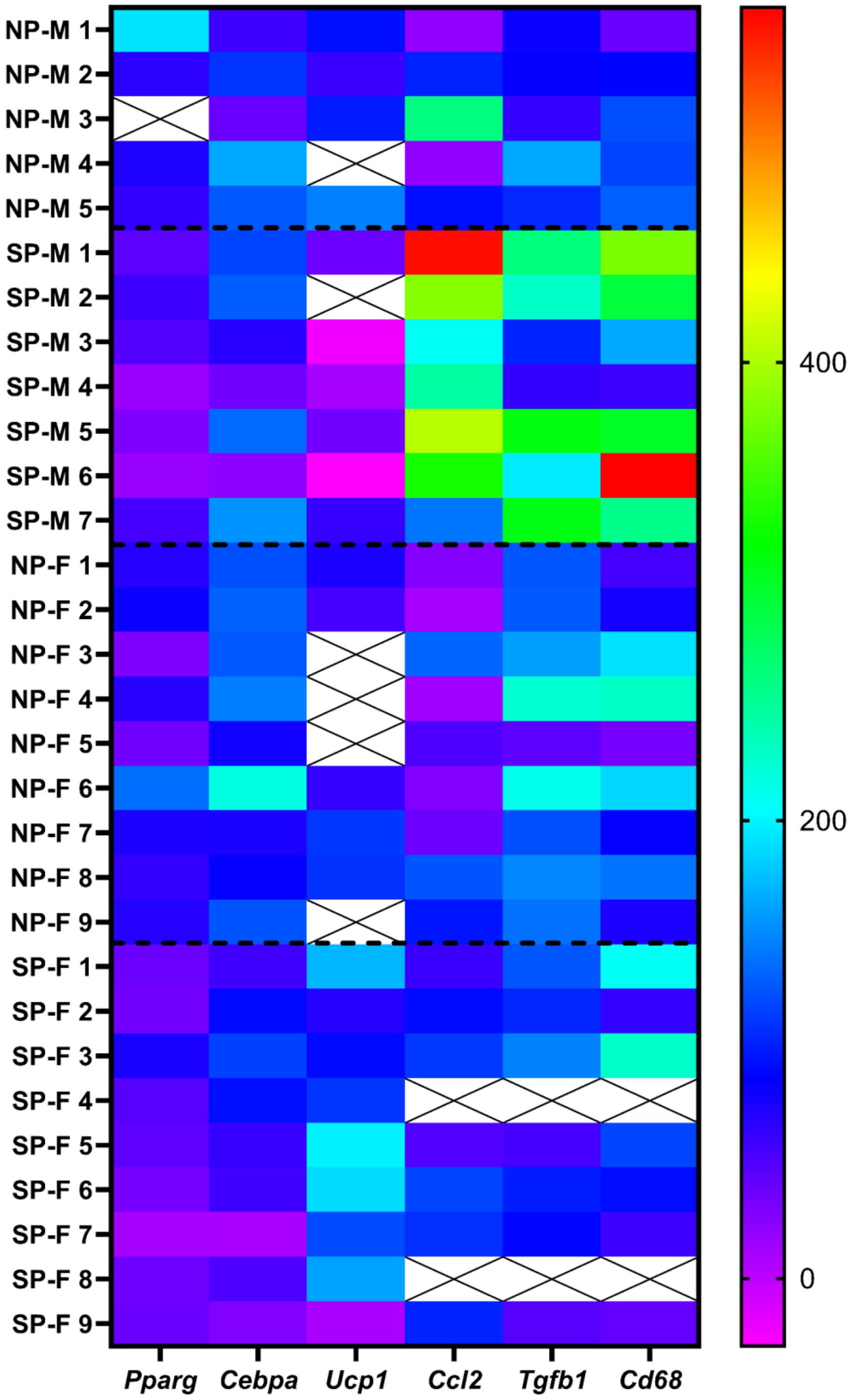

Supplemental Figure S4: Differential visceral gene changes with short photoperiod exposure in males and females. Male and female *P. obesus* were exposed to neutral (12 hr light:12 hr dark) or short (SP, 5 hr light:19 hr dark) photoperiods and a high energy diet for 20 weeks. Expression heatmap showing differential visceral expression of *Pparg*, *Cebpa*, *Ucp1, Ccl2*, *Tgfb1*, and *Cd68* mRNA. NP-M: neutral photoperiod males, SP-M: short photoperiod males, NP-F: neutral photoperiod females, SP-F: short photoperiod females.

Supplemental Figure S5

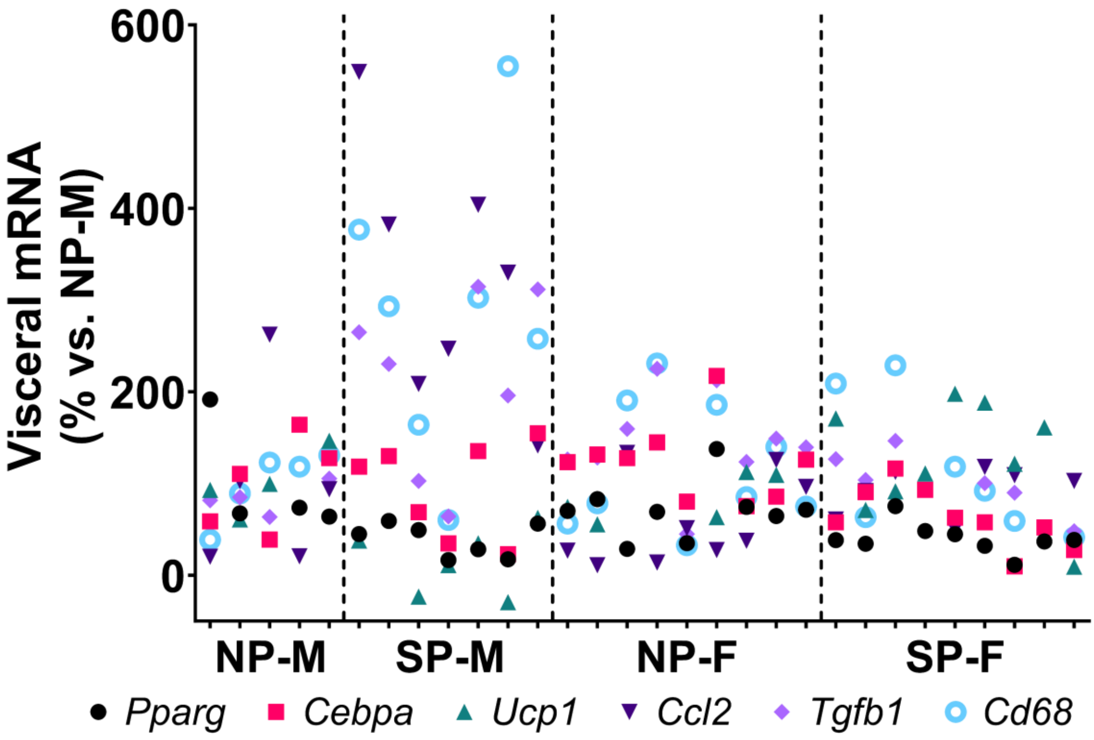

Supplemental Figure S5: Differential visceral gene changes with short photoperiod exposure in males and females. Male and female *P. obesus* were exposed to neutral (12 hr light:12 hr dark) or short (SP, 5 hr light:19 hr dark) photoperiods and a high energy diet for 20 weeks. XY plot showing differential visceral expression of *Pparg*, *Cebpa*, *Ucp1*, *Ccl2*, *Tgfb1* and *Cd68* mRNA. NP-M: neutral photoperiod males, SP-M: short photoperiod males, NP-F: neutral photoperiod females, SP-F: short photoperiod females.

Supplemental Figure S6

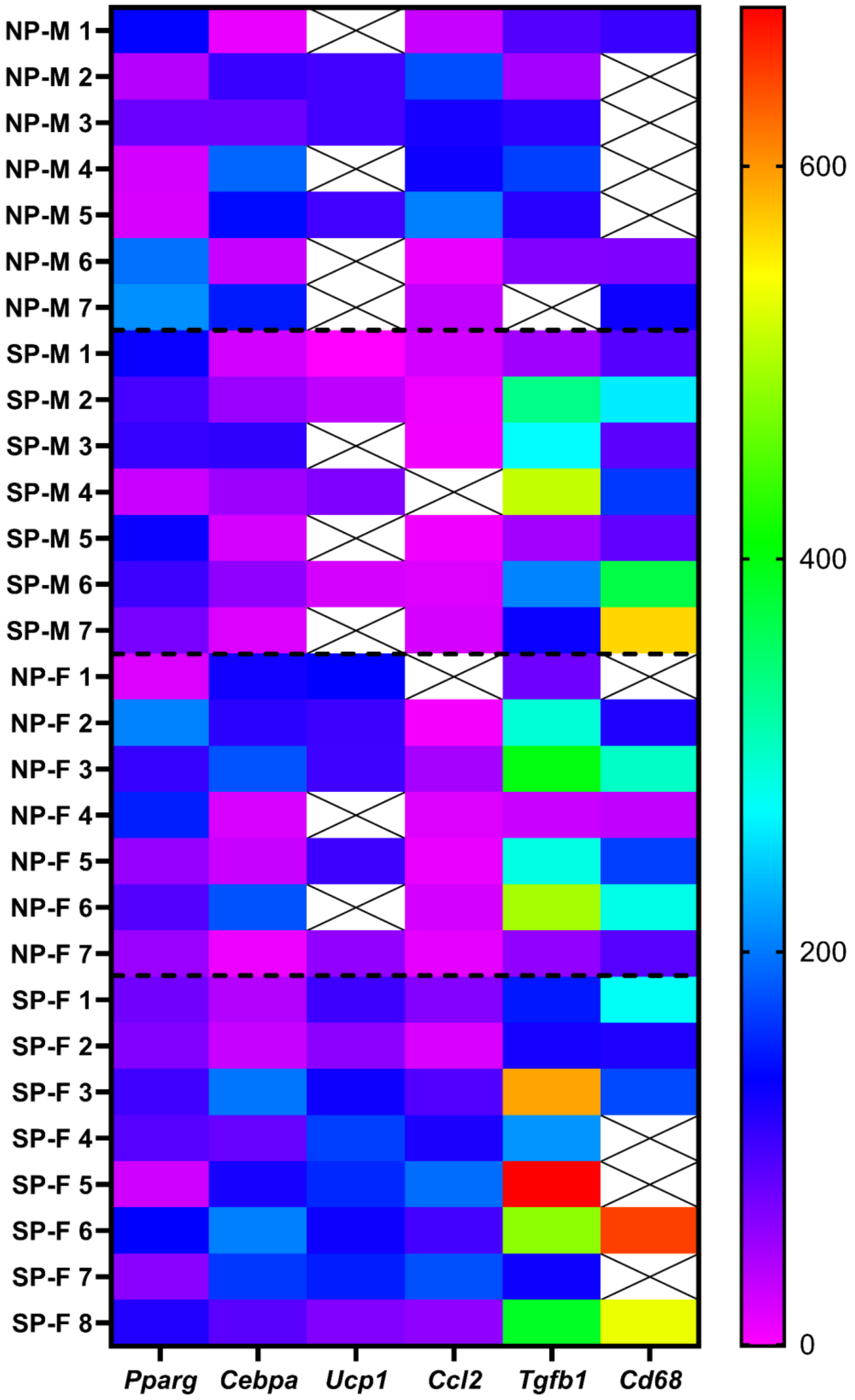

Supplemental Figure S6: Differential subcutaneous gene changes with short photoperiod exposure in males and females. Male and female *P. obesus* were exposed to neutral (12 hr light:12 hr dark) or short (SP, 5 hr light:19 hr dark) photoperiods and a high energy diet for 20 weeks. Expression heatmap showing differential subcutaneous expression of *Pparg*, *Cebpa*, *Ucp1, Ccl2*, *Tgfb1*, and *Cd68* mRNA. NP-M: neutral photoperiod males, SP-M: short photoperiod males, NP-F: neutral photoperiod females, SP-F: short photoperiod [illegible]

Supplemental Figure S7

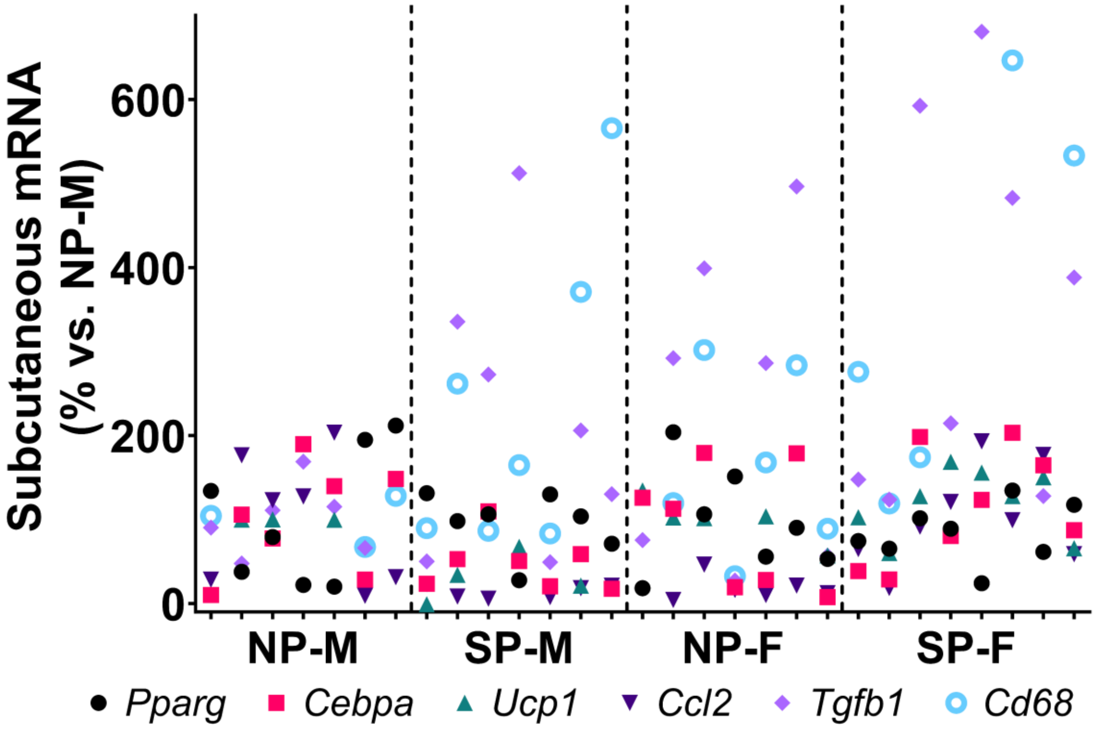

Supplemental Figure S7: Differential subcutaneous gene changes with short photoperiod exposure in males and females. Male and female *P. obesus* were exposed to neutral (12 hr light:12 hr dark) or short (SP, 5 hr light:19 hr dark) photoperiods and a high energy diet for 20 weeks. XY plot showing differential subcutaneous expression of *Pparg*, *Cebpa*, *Ucp1*, *Ccl2*, *Tgfb1* and *Cd68* mRNA. NP-M: neutral photoperiod males, SP-M: short photoperiod males, NP-F: neutral photoperiod females, SP-F: short photoperiod females.
